# Supplementary material for: Are carbon emissions trading and green financial instruments synergistic? -Comprehensive quantitative research based on content analysis
Source: PLoS One. 2024 Mar 7;19(3):e0298601. doi: 10.1371/journal.pone.0298601 (PMC10919601; doi:10.1371/journal.pone.0298601)
Supplement: S1 Data — (ZIP) [file pone.0298601.s001.zip › Data/地级市Z_F工作报告.zip!/╣π╬≈/║╪╓▌╩╨/└┤╫╘╣1⁄2╓┌║┼í╛┬φ┐╦╩2╛▌═°í┐_2009.docx]

各位代表：

现在，我代表市人民政府向大会作工作报告，请予审议，并请市政协各位委员和其他列席会议的同志提出意见。

一、2008年工作回顾

2008年是极不寻常的一年。我们在自治区党委、政府和市委的正确领导下，在市人大、市政协的监督支持下，紧紧依靠全市人民，深入贯彻落实科学发展观，着力实施“科学发展突破发展”三年计划，积极应对国内外复杂经济形势和冰雪洪涝灾害的严峻挑战，经济社会平稳发展。全市实现生产总值252.8亿元, 增长6.4％;财政收入16.01亿元，增长1.2％，其中税收增长20.2%，占财税收入比重达75%;全社会固定资产投资159.2亿元，增长19.1％;城镇居民人均可支配收入12772元，增长15.4%；农民人均纯收入3458元，增长11.8%。

三次产业协调发展。产业结构进一步优化，三次产业分别增长3.9%、6.1%和8.8%,三次产业比重调整为21.7∶52.5∶25.8。工业主导地位进一步增强。实现工业增加值112.5亿元，增长6%，其中规模以上工业实现增加值42.2亿元，增长1.1%。工业增加值占生产总值比重44.5%，对经济增长的贡献率39.2%。完成工业固定资产投资73.3亿元，增长25.9%。其中，建设技改项目136个，完成技改投资15.6亿元。园区规划建设取得新成效，拥有1个省级、3个A类、2个B类工业园区，成为全区唯一实现产业园区全覆盖的地级市。新农村建设扎实推进。粮食总产量增长1.1%，经济作物播种面积增长5.6%，建成广西最大的脐橙生产基地。蔬菜、茶叶、优质粮、食用菌等优势农产品种植面积扩大6.2万亩,发展订单农业 43.2万亩。新增规模农业企业35家。发放水稻良种补贴等五项直补资金1.25亿元，投入水利工程资金3.8亿元。完成造林面积27.7 万亩，增加22.4％。以商贸旅游业为龙头的第三产业平稳发展。黄姚古镇被评为国家4A级旅游景区和中国历史文化名镇，十八水原生态公园被评为国家3A级旅游景区。全市接待游客370.7万人次，增长13.3 %；旅游总收入22.1亿元，增长21.8 %。继续抓好各类专业市场和商贸设施建设，深入实施“万村千乡市场工程”，激活了农村消费市场。社会消费品零售总额58.4亿元，增长23.9%。外贸出口总额9581万美元，增长10.3%。

城市面貌明显改观。完成市区“一江两岸”景观规划，编制了火车站片区控制性详细规划，完成太原小区、贺州大酒店、大修厂住宅区等26个项目的修建性详细规划的编制和审批。“一江两岸三新区”、汽车客运中心、生活垃圾无害化处理厂二期工程、贺州学院新校区、市职业教育中心等重点市政项目建设进展顺利,市污水处理厂项目抓紧实施,城区交通、管网、绿化、亮化、供水、人防等市政公共基础设施建设顺利推进。“城乡清洁工程”继续深入实施，城市管理综合执法得到加强。创自治区文明城市工作取得成效。

项目建设取得重大进展。在建投资超过亿元的项目35个,项目投资拉动GDP增长2个百分点以上。一批重点项目建设步伐加快，桂梧高速公路同古至潮江段基本建成，洛湛铁路贺州段完成铺轨;贵广快速铁路已开工建设;广贺高速公路贺州段建设速度加快；2008年列入自治区层面统筹推进的华润水泥厂、贺州市木材(家具)加工集散基地及大桂山林场50万立方米人造板生产线等6个新开工项目全部实现开工。华润电力贺州电厂工程拿到了国家发改委的“路条”,一批重点争取和做前期工作的项目取得了显著成果。在项目资金争取上，全年落实中央预算内和国债资金2.6亿元，超过“十一五”计划前两年总和。

招商引资工作有新成效。加快旺高、西湾（平桂）、信都工业区和各县（区、管理区）工业集中区等承接产业转移平台的建设，组团参加第四届港澳广西活动周活动、第五届中国—东盟博览会和中国—东盟商务与投资峰会，积极做好承接东部产业转移工作。全市新签招商引资项目的投资总额和实际到位资金持续快速增长，全年新签外来投资项目715个，投资总额225.3亿元，增长17.8%。外来投资项目（含续建）到位资金124.8亿元，增长19.2%，其中外资实施项目到位资金3820万美元，增长63.5%。

民生得到保障和改善。全力做好上半年遭遇的百年罕见的低温雨雪冰冻灾害和较严重的洪涝灾害的防灾救灾工作,因灾倒塌房屋和损毁基础设施重建工作顺利完成，取得抗击灾害的全面胜利。组织发动全市向四川汶川地震灾区捐赠款物1159万元。千方百计保障就业，全市城镇新增就业11374人,其中城镇下岗失业人员再就业3853人,分别完成任务的113.7%和137.6%，城镇登记失业率控制在4%以内。社会保险覆盖面进一步扩大,五项社会保险参保30万人次。建立廉租住房制度,解决了284户最低收入家庭住房困难问题。支出保障金1.01亿元,保障城市居民低保对象42846人、农村低保对象93351人。第二批40个整村推进扶贫任务顺利完成。新建沼气池19725座、农村人畜饮水安全工程87处。

为民承诺十件实事如期完成。八达西路破损路面维修工程和207国道贺州段、301省道信都至灵峰段二级公路水泥砼路面主体工程完工并投入使用；资助78238名贫困学生上学,实现了“不让一个学生因家庭贫困而失学”目标;改造中小学校食堂,成为全区第二个全部实现中小学校食堂量化分级管理C级以上标准的地级市; 严历打击“两抢一盗”犯罪行为，案件破案829起,破案数上升；关停13家限期治理不达标的排污企业，龟石水库库区生态环境持续改善；顺利开展非法营运三轮车整治工作，进一步完善市区公交系统设施；农村特困户危房改造完成率174.8%；全市新型农村合作医疗实现全覆盖，参合率85.5%；在市区建成2所社区卫生服务中心，社区卫生服务覆盖市区人口达70%；清理整治非法采矿矿点180多个，炸毁非法淘砂船22艘,行政处罚70多人次，有效遏制非法采矿行为；市区公共卫生设施建设任务顺利完成。

社会事业取得新进步。 全面实施第四轮科技创新计划，推广应用先进实用技术42项。素质教育扎实推进，荣获全国青少年航空航天车辆模型总决赛和全国第23届青少年科技创新大赛金奖。“两基”成果继续得到巩固和提高，农村留守儿童教育机制有创新，中小学校教学装备水平取得新突破，全市城乡义务教育阶段学生全部免除学杂费，农村义务教育阶段学生和城市低保家庭学生享用免费教科书，职业教育攻坚全面实施，其他各级各类教育取得显著成绩。竞技体育和全民健身运动取得新成效。新闻出版业持续健康发展，出版物市场监管和知识产权保护进一步加强。文化事业发展加快，文化基础设施建设不断加强,率先在全区实现农村电影放映数字化。公共卫生体系和基本医疗服务体系不断完善，城市社区卫生服务获得新发展。人口和计划生育工作取得新成绩，人口自然增长率控制在9‰以内。广播电视“村村通”工程建设加快，农村广播电视无线覆盖率65%。节能减排工作扎实开展，年度淘汰落后产能工作任务按时完成。市本级、各县污水处理厂项目抓紧实施。“贺州清新”行动扎实推进，生态环境不断改善。社会治安综合治理和平安贺州创建活动取得新成效。普法工作有序开展，人民调解工作成效显著。积极开展大接访活动，市领导接待群众来访195批，立案130件，办结率100%。成功化解三轮车整治、征地拆迁、企业关闭破产所衍生的大量矛盾，维护了社会稳定。产品质量和食品药品监管进一步加强，安全生产继续好转。国防后备力量建设深入开展，双拥工作取得新成绩。

一年来，我们以开展继续解放思想大讨论活动和深入学习实践科学发展观活动为契机，不断加强政府自身建设。认真执行市人大及其常委会各项决议决定，自觉接受人大及其常委会的法律监督和工作监督。加强同人民政协的联系，支持政协委员发挥参政议政作用。广泛听取各民主党派、工商联、无党派人士意见，支持工会、共青团、妇联等人民团体更好发挥作用。进一步转变政府职能，健全政府新闻发言人制度，实行了重大事项公示和听证制度，加强了政府工作督查。继续精简和规范行政审批，拓宽政务公开渠道，加强政务服务中心建设和管理，深化政府采购招投标体制改革。加强政府法制建设，严格依法行政，强化廉政建设，提高政府的执行力、控制力和公信力。此外，外事、侨务、民族、宗教、监察、人事、机构编制、审计、统计、物价、工商、技术监督、检验检疫、供销、地震、气象、地方志、档案、老龄、残疾人、住房公积金等工作取得新进步。

各位代表！回顾2008年的工作，成绩来之不易。这是全市各级各部门和210多万各族人民团结奋斗，攻坚克难，开拓进取的结果。在此，我代表市人民政府，向全市人民，向驻贺部队、武警官兵和公安干警，向各民主党派、工商联、无党派人士、人民团体以及所有关心、支持贺州建设和发展的各界人士，表示衷心的感谢和崇高的敬意！

回顾一年来的工作，我们清醒地看到，我市经济社会发展中还存在不少困难和问题：由于国际金融危机持续蔓延等多种原因，我市经济增速出现明显回落；部分行业企业生产经营困难，经济效益下滑；农业受灾严重，农村基础薄弱，统筹城乡发展面临困难增多；劳动就业、社会保障、教育、医疗卫生、安全生产等涉及群众切身利益方面还有不少问题需要认真加以解决，社会稳定压力还比较大；政府工作与人民的期望还有差距，等等。我们一定高度重视这些困难和问题，以对党对人民高度负责的使命感和紧迫感，更加沉着应对，更加努力奋斗，绝不辜负人民的重托。

二、2009年工作总体要求和奋斗目标

中央和自治区认为，今年是新世纪以来我国、自治区经济发展最为困难的一年，也是蕴含重大机遇的一年。我市情况与宏观形势完全一致。尤其是我市产业结构层次低、企业素质总体不高、市场竞争力较弱，经济发展面临的挑战非常严峻。虽然我们遇到了前所未有的困难，但也蕴含着很多加快发展的有利条件，特别是国家近期出台的扩大内需促进增长的政策，必将给我市带来加快发展的广阔空间。另外，通过建市六年来的不懈奋斗，我市发展的基础日渐厚实，交通大通道格局初露端倪，投资环境日趋优化，一批重大基础设施和产业项目将陆续开工建设或建成投产，为我们争取更多的国家投资创造了良好的条件。“信心比黄金重要”。我们既要增强忧患意识，更要增强机遇意识，注重从变化的形势中捕捉和把握发展机遇，增强战胜困难的信心和决心，以非常办法、非常措施、非常力度、非常政策，化压力为动力，化挑战为机遇，化危机为转机，奋力推进我市经济社会持续快速发展。

2009年政府工作的总体要求是：以邓小平理论和“三个代表”重要思想为指导，深入贯彻科学发展观，全面落实党的十七大、十七届三中全会精神和市委二届五次全会精神，以突破发展为首要任务，以投资拉动为保障，以扩大内需为基础，以调整结构为主线，以改善民生、构建和谐为根本，全力开展重点项目攻坚，大力推进工业、交通、城建、旅游突破发展，夯实农业基础地位，为加快建设富裕和谐新贺州而努力奋斗。

今年经济社会发展的主要预期目标为：生产总值增长11%；财政收入增长10%；全社会固定资产投资增长25%；社会消费品零售总额增长14%；单位生产总值能耗降低2.3%， 二氧化硫排放总量控制在3.2万吨以内，化学需氧量排放总量控制在3.15万吨以内；外贸进出口总额增长10%；城镇居民人均可支配收入增长12%；农民人均纯收入增长10%；城镇新增就业1.2万人；人口自然增长率控制在9‰以内。上述目标中，人口自然增长率、万元生产总值能耗、主要污染物排放量是约束性指标，必须想方设法完成；其他指标是预期性的、指导性的，在实际工作中，我们将朝更高目标努力。

三、2009年主要任务

实现今年经济社会发展的各项目标，必须以非常措施、过硬手段、超强力度，重点抓好以下八项工作：

（一）抢抓国家宏观调控机遇，千方百计扩大投资规模

全面把握中央扩大内需促进经济平稳较快增长的各项政策，全力开展重点项目攻坚，组织实施365个重点项目建设，保持投资对经济增长的强劲拉动。

强力推进重大项目建设。抓好新开工项目，全力争取华润电力贺州电厂一期工程项目获国家审批核准实现开工；尽快衔接计划,完善开工手续，落实建设资金,尽早实现永贺高速公路贺州段、贺州学院二期工程、贺州油库、八步区灵峰标准厂房建设、钟山县邝氏矿业有限公司陶瓷原料加工生产等项目开工建设,形成新的投资量。抓好续建项目投产达产工作，尽快实现桂梧高速公路、广贺高速公路贺州段建成通车；华润水泥厂、上程电站、大陆亿康食品加工、市职业教育实训基地、市木材(家具)加工集散基地及大桂山林场50万立方米人造板生产线等续建重大项目投产达产。加快推进柳州经贺州至广东韶关铁路贺州段、支线机场、林浆纸一体化、风力发电场等项目前期工作,力争早日立项。

积极争取资金落实。积极争取上级资金和银行信贷支持。充分利用好国家实施积极的财政政策和适当宽松货币政策的机遇，及时向自治区和国家有关部门衔接汇报，争取更多上级资金支持；紧紧抓住今年全区商业银行增加2000亿贷款的好机会，积极协调银企关系，帮助企业落实贷款条件，促进银行对重点建设项目的信贷扶持，并充分利用城投公司、交投公司、旅投公司等投融资平台，努力争取项目信贷资金。积极推动企业直接融资。加快中小企业的融资体系建设，加强对现有投融资担保企业的指导，定期召开金融部门与中小企业见面会，随时掌握中小企业资金需求情况，为扩大中小企业融资规模做好服务。积极落实地方财政配套资金。依法依规利用好民间资金。

着力解决项目征地拆迁问题。依法做好征地报批，千方百计争取用地指标；依法清理盘活闲置土地，调整、整合存量国有土地，优先满足重点项目用地需求。加大征地拆迁实施力度，城区征地拆迁工作实行以市政府统一领导、县（区、管理区）政府（管委）按辖区负责、市直有关部门依职能配合的工作运行机制，层层落实责任。依法依规做好征地拆迁工作，加大对群众的思想教育和宣传，把有关补偿标准、安置政策落实到位，争取群众支持，确保项目建设不因征地拆迁问题而受到影响。

超强抓好重点项目前期工作。加快项目报批速度，做好项目用地、规划、环保手续的报批等方面的跟踪衔接工作。尤其要加快征地报批，争取符合条件的项目列入自治区重点项目盘子，努力解决建设项目用地指标问题。用好用足自治区下放我市的审批核准权限，优化审批核准程序，减少审批核准手续，确保项目审批中环节少、速度快、效率高。加大项目前期工作经费投入。今年市本级财政安排2000万元作为项目前期工作经费，各县（区、管理区）安排500万元以上。同时，引导有关企业、各项目业主加大前期经费投入，创造机会上新项目。积极做好项目储备和年度项目前期工作推进计划，力争项目储备规模达到2500亿元以上。尤其要抓住国家实施产业振兴规划和自治区财政安排工业产业发展资金10亿元支持全区重点技改项目和工业园区基础设施建设的有利机遇，围绕企业异地改造搬迁、关小上大、节能减排、延长产业链、园区建设等方向抓好技改项目申报，积极争取更多的技改项目纳入国家和自治区发展规划。

切实加强对项目工作的组织领导。充分发挥市重点项目建设指挥部的组织协调作用，加强对重点项目的跟踪服务。按照“一个项目、一个联系领导、一个责任单位、一个工作班子、一个倒排工期表”原则，把项目服务责任落实到单位和个人。狠抓项目报批、征地拆迁、资金落实和建设进度，严格实行重点项目推进任务考核和奖惩制度，加快推进重点项目建设。

（二）强化产业支撑，壮大工业经济实力

以重大项目带动产业优化升级，促进工业做大做强。年内工业固定资产投资增长50%以上，工业增加值增长12%。

抓服务，帮助企业解困。扎实开展“服务企业年”活动，强化运行服务保障。加强工业运行分析监测，健全煤电油运和重要原辅材料调度机制，在产品营销、运输调度、劳动保障等方面帮助困难企业渡过难关。协助企业化解融资难问题、减轻税费负担。 鼓励和支持非公有资本进入法律法规未禁止进入的基础设施、公共事业及其他行业和领域。破除体制障碍，坚持平等保护物权，推进公平准入，形成各种所有制平等竞争、相互促进的新格局。

抓技改，增强企业竞争力。引导和扶持现有骨干企业加快技术改造，实施总投资113.9亿元的60个重点技改项目，力争年内完成投资46.6亿元。帮助企业积极争取上级专项、贴息、扶持、奖励等资金，用于企业技改或创新。同时，加快以企业为主体的技术创新体系建设，不断提升自主品牌的竞争力和影响力。重点加快建设科技企业孵化中心，培育国家级、省级企业技术中心、技术检测中心。

抓骨干，做强支柱产业。主攻电力、林产、有色冶金、电子、食品制药、建材等六大优势产业，优化产业结构。确保贺州南华糖厂、吉光电子电解电容器、珊瑚钨矿采选、隆德粉体超细粉、宏鑫化工硫铁矿制酸等项目年内投产；实现华润电力贺州电厂一期工程、桂东电子三期工程、华润水泥配套余热发电厂、金源稀土材料产业化、广西有色集团矿产加工等一批重点项目开工建设。力争年内六大优势产业规模以上企业实现总产值112亿元以上。积极实施中小企业成长工程，力争全市工业中小企业户数达500户。抓好中小企业创业基地、农产品加工创业基地建设，构建良好的创业平台。

抓园区，打造工业发展平台。重点抓好旺高、平桂（西湾）、信都工业区规划建设，着力推进电子科技工业园规划与建设，加强各县（区、管理区）工业集中区建设，力争年内全市各类工业园区完成基础设施投资2亿元，增长20%。围绕工业园区开展招商引资，充分发挥工业园区的集聚优势，力争年内旺高、平桂（西湾）、信都工业园区共实现工业增加值23.7亿元，税金2.65亿元。

抓节能减排，促进产业优化升级。强化节能减排目标责任制和“一票否决制”。督促落实单位GDP能耗下降目标任务、规模工业增加值能耗下降任务、全市7家重点耗能企业实现节能目标任务。实施适用节能技术研发和推广等节能及污染物减排重点工程，加强污染物减排监管，推动全市工业企业节能减排工作，促进产业结构升级。

（三）加快推进城镇化进程，提升城市综合竞争力

围绕打造“森林之城、田园都市”目标，全面提升城市综合素质和竞争力，实现城市发展新突破。

完善城镇规划体系。完成修编新一轮贺州市城市总体规划及贺州大道（南段）周边地区、新兴街西片旧城区等一批控制性详细规划，力争实现城市控制性详细规划全覆盖。组织编制贺街镇总体规划和临贺故城保护规划、市绿地系统专项规划和绿地系统防灾规划。

加强城市建设和管理。坚持把城市建设作为拉动投资和内需的重要渠道,加快城建重点项目建设,提高城市品位。重点加快“一江两岸”建设。搞好以“堤路园”（江南段）为先导的江南沿岸建设；完善江北中路、江北东路人行道、绿化等配套设施建设。努力推进火车站新区、江南新区、城东新区的建设。完成光明大道、火车站站前路至平桂管理区一级路、八达西路延长线等项目的规定工作量；抓紧推进铁路物流中心、汽车客运中心、职业教育中心等项目建设；加快灵峰南路、广贺高速东连线等项目建设。年内建成市污水处理厂。继续深入实施“城乡清洁工程”，加强城市综合管理，确保市容整洁有序，绿化、亮化、美化水平有新提高，发展环境和人居环境进一步优化。加强土地储备工作，依法严格管控土地一级市场，为加快城市发展提供土地保障。

加快县城和重点镇建设。加快各县县城垃圾填埋、污水处理等市政基础和公用设施建设。积极推进钟山县滨江花园商住区、河东商贸城等“六大市政工程”，富川县城东摩托车专业市场、凤凰景园商住小区，昭平县城区防洪排涝工程、茶叶宝石街、天鹅塘经济适用房小区，平桂管理区行政中心、平桂大道、望高火车站站前广场，八步区桂岭商住中心、信都江滨小区、贺街辰泰临贺国际城等重点城镇建设项目。

（四）推进农村改革发展，保持农民收入稳定增长

全面贯彻党的十七届三中全会和中央农村工作会议精神，深入推进农村改革发展，加快社会主义新农村建设，努力增加农民收入。

以改革增添活力。积极推动农村土地承包经营权“依法、自愿、有偿”流转；加快实施农村社区股份合作、土地股份合作和专业合作改革；积极稳妥地推进集体林权制度改革试点工作；深化农村信用社改革，鼓励发展小额信贷。健全社会主义新农村建设驻村指导员、科技特派员制度。加快建立新型农技推广服务网络。培育有文化、懂技术、会经营的新型农民，发挥广大农民在新农村建设中的主体作用。

以稳粮保障供给。落实粮食安全行政首长负责制。大力推广新品种新技术，稳定发展粮食生产，确保粮食安全。继续提高粮食最低收购价格，落实粮食直补、良种补贴、农资综合直补和农机购置补贴等政策，确保种粮农民得到实惠。科学制定并严格实施土地利用总体规划，建立对外耕地保护责任区目标责任制，坚持占用耕地补偿制度，切实节约用地。

以结构调整促进增收。深入开展“优果工程”,加快推进脐橙、蔬菜、马蹄、茶叶、烟叶等传统特色产业区域化布局和发展规划,力争优势农产品种植面积扩大6－8万亩,年内创建“一村一品”专业示范村屯10个以上。加快无公害产地认定和“三品”产品认证步伐，力争全年新增产地认定面积15万亩以上,新增产品认证、农产品注册商标各10个,申报全国、广西名优农产品3－5个。积极筹办好第五届贺州农交会。加大农业龙头企业培育力度，力争新增农业规模企业30家,新增自治区级龙头企业1家以上。加快养殖业结构调整,着力推进水产畜牧规模化、标准化和产业化经营。建设农产品质量安全监管体系,健全重大动植物疫病防控体系。

以多予夯实基础。继续加大公共财政向“三农”倾斜力度，拓宽农业投入来源渠道。全民动员，开展水利建设大会战，完成10座中型和重点小型病险水库的除险加固。推进农村路网建设，修建通达工程25项230公里、通村水泥路163公里。抓好农村生态能源建设，力争完成建设1.5万座沼气池。加快实施“千乡万村现代农机装备推进工程”。坚持开发式扶贫方针，启动实施第三批41个贫困村整村推进扶贫开发项目。

（五）积极培育消费热点，努力扩大消费需求

加大政策扶持力度，积极扩大消费,努力增强消费对经济增长的拉动作用。

努力扩大城乡消费。继续推进“万村千乡市场”、“双百市场”和“农村现代流通网络”工程建设，推动现代流通方式向农村延伸,大力促进农民消费；做好“家电下乡”、“农机下乡”工作，扩大农村市场规模。发展城市社区商业，营造多元化、多层次的城市居民消费服务网络；合理引导文化、健身、娱乐、休闲等消费。认真落实产品质量安全责任制，强化产品质量和食品药品安全监管；加强粮食、食用油、肉类等基本生活必需品的生产、调运和储备，保障市场供给，加强价格监管，保持物价基本稳定。

稳定发展房地产业。认真落实国家出台的房地产政策及《促进贺州市城市建设暂行办法》，加大居民购房政策支持力度，落实和完善促进住房消费的财税、信贷政策。从土地、金融、税收、行业管理等方面加大对房地产企业的扶持，提高企业抗风险能力。加大房地产市场监管力度，维护房地产市场秩序。增强市场信心，稳定市场预期，引导合理的房地产投资和住房消费。鼓励房地产开发向县城和重点镇延伸。坚持农民住房建设与新农村建设相结合，加大公共财政投入，加强村（屯）规划和建房技术服务，鼓励农民拆旧建新，促进农村住房消费。

突破发展特色旅游业。全面推进乡村旅游示范点建设,形成一定规模和产业体系；完善旅游项目规划建设,成立市旅投公司并正式运营，策划启动桂台客家文化旅游贺州实验区、钟山十里画廊景区、姑婆山国家森林公园休闲度假区、黄姚国家历史文化名镇旅游基础设施建设等项目,加快推进大桂山风景旅游度假区等续建项目建设；加强旅游基础设施与配套产业建设,启动紫云洞景区道路建设工程、八步至姑婆山旅游区专线公路扩建工程。扎实推进贺州、桂林两市的旅游产业发展战略合作,深入实施“两广六市”旅游交流与合作,开展形式多样的宣传促销活动,提高旅游知名度。力争全市接待国内外游客420万人次,实现旅游总收入27亿元。

加快发展相关服务业。加快推动传统商贸服务业转型升级。加快金泰农产品物流加工项目、金旗小五金市场、湖广大市场、石凉亭农贸市场、羊角山农贸市场、厦良农贸市场、建材市场等一批市场建设。大力发展交通运输、现代物流、金融保险、信息咨询等生产性服务业，加快发展市政公用、社区服务、中介等生活性服务业。

（六）全方位开展招商引资，努力提升对外开放水平

坚持外引内联并举原则，加大招商引资力度，全力推动外向型经济快速发展。

加强多区域合作。以工业园区为载体，以重点企业为主体，以专业队伍为骨干，以中介组织为辅助，有针对性地开展产业招商、专业招商、以商招商、会展招商、小分队招商。着重融入粤港澳，积极拓展闽苏浙，主动挺进东南亚，全面加强与周边城市在交通、能源、矿产开发、旅游、农业等领域的合作。尤其要加强贺州、梧州、玉林、贵港四市承接东部产业转移联运协作，有的放矢地制定承接方案，出台更加优惠的政策，拿出好的项目，与国内外大企业搞好对接，打造“创业贺州”区域经济合作平台，着力建设承接东部产业转移基地。同时，加强“央企入桂”、“百企入桂”工作。

积极推进招商引资大兑现工作。建立和完善各级招商引资大兑现项目库，提高项目审批代办服务水平，抓好项目推进跟踪协调服务工作，加强项目的督查力度，提高项目的“合同履约率、资金到位率、开工竣工率”，提高利用外资的规模和水平。力争全年招商引资区外境内项目到位资金140亿元，实际利用外资到位资金5160万美元。

着力优化投资软环境。完善投资优惠政策，打造成本洼地。进一步完善和落实招商引资奖励政策。深化效能建设，创新服务机制，抓好项目审批、协调督办机制创新和制度建设，提高招商推进项目协调工作效率。抓好外来投资投诉受理和协调工作，不断完善投资软环境。

确保外贸出口平稳较快增长。着力落实国家和自治区支持出口的有关政策，帮助企业降低出口运营成本，拓宽出口融资渠道，增强出口创汇盈利能力和市场竞争能力，培育和扩大外贸出口队伍。积极承接加工贸易产业转移，加快发展加工贸易。确保进出口总额增长10%以上。

（七）抓好财税工作，强化保障支撑

审时度势，未雨绸缪，加强财税工作，构建保障有力的公共财政服务体系。

狠抓财源培育。全面贯彻落实积极的财政政策，争取上级财政更多的支持。针对国家全面实施增值税改革、资源税改革以及税费政策调整的新情况，积极发挥财政职能作用，扶持税收主体和骨干税源，支持重点财源项目加快建设和发展，培植一批税收超千万元的纳税大户，促进税收持续较快增长, 努力提高税收占财政收入的比重。坚持依法治税，科学征管，打击各种涉税违法行为，确保应收尽收。规范非税收入征缴和管理，拓宽财政增收渠道。

加强财政预算管理。按照控制非生产性支出，增加扩大内需、促进经济增长支出的原则，调整优化支出结构，增加社会保障、教育、卫生等方面支出，加大对农业和农村发展、自主创新、能源资源节约、生态环保以及主导产业等重点领域的投入，保障经济社会发展薄弱环节和公共服务支出需要。严格控制一般性支出，对公务购车用车、会议经费、公务招待费用、出国（境）经费等支出实行零增长，减少行政成本。严肃财经纪律，严格执行国库集中收付和政府采购制度、财政投资评审制度，加强公务支出资金的监管，提高资金使用效益。规范行政事业单位占有、使用、处置国有资产行为，加强政府债务管理，提高科学理财水平。

（八）切实保障和改善民生，加快发展社会事业

坚持从群众根本利益出发，加大投入，改善民生，促进各项社会事业全面进步。

实施积极的就业政策。开展全民创业活动，健全创业服务体系，完善支持自主创业和自谋职业的各项政策，以创业带动就业。积极帮助下岗失业人员再就业、“零就业”家庭和就业困难人员就业。加强对高校毕业生就业的指导和服务，高度重视退伍转业军人、营运“三轮车”车主及残疾人就业问题。加强对城镇下岗失业人员再就业、农民工技能和农村中学毕业生的培训。落实就业促进法和鼓励创业的税收优惠、小额担保贷款、资金补贴等政策措施，落实职业介绍、职业培训、社会保险和公益性岗位补贴政策。推进统筹城乡就业，年内新增城镇就业1.2万人，城镇登记失业率控制在4%以内，新增农村劳动力转移就业3万人。

完善社会保障体系。加快建立覆盖城乡的多层次社会保障体系，加强社会保障基金的征缴、管理，完善各类社会保障的政策衔接，继续做好“两个确保”和城乡低保工作，继续抓好养老、失业、医疗、工伤和生育等保险的扩面征缴，不断提高保障水平。进一步完善新型农村合作医疗制度，力争农民参合率达90%以上。稳步提高低收入人群收入。抓好廉租住房和经济适用住房建设，着重推进市鸡笼山廉租房项目建设和江南新区经济适用住房小区建设。

统筹发展各项社会事业。深入实施第四轮科技创新计划，完善科技培训体系，广泛开展科普活动。加强创新型人才和各类紧缺人才的培养、选拔和引进，促进人才资源的合理配置。优先发展教育事业，加大教育基础设施建设投入，抓好农村中小学校校舍维修改造、教职工安居工程、中小学校饮水安全工程和卫生厕所建设等工作，加大农村中小学校师资培训力度。理顺城区义务教育管理体制，加快规划和建设城区义务教育学校。适当扩大高中办学规模，创建自治区示范性高中。加大职业教育攻坚力度，加快驻梧中专学校整体搬迁贺州步伐。推进贺州学院建设，提高高等教育质量，全面落实国家生源地信用助学贷款政策，做好贫困生资助工作。广泛开展群众性精神文明创建活动和全民健身运动，提高竞技体育运动水平。抓紧推进贺州大钟山转播台无线覆盖工程建设，规划建设20户以上“村村通”广播电视村点1040个。启动市文化会展中心建设，全力推进农村电影放映、农家书屋、文化信息资源共享和乡镇综合文化站等公共文化项目建设，进一步加强新闻出版工作，加大知识产权保护力度，深入开展“扫黄打非”。重视非物质文化遗产挖掘保护，做好全国第三次文物普查工作。稳步推进医药卫生体制改革，加快建立城市社区卫生服务网络，加强农村三级卫生服务网络建设。认真落实人口与计划生育法律、法规政策，全面实现工作重心下移，强化层级责任，建立完善以村为主的工作机制，大力推进村级绩效管理。深入开展创建全国计划生育优质服务先进县（区）活动， 扎实推进全国婚育新风进万家活动示范市建设，加强流动人口计生服务和管理，稳定低生育水平，提高出生人口素质。充分发挥工会、共青团、妇联等群团组织的桥梁纽带作用。促进红十字会、老龄、残疾人、监察、机构编制、工商、技术监督、统计、人防、气象、外事、侨务、对台、供销、档案、地方志、技术监督、检验检疫、住房公积金等工作不断发展。

加强社会管理。加强基层政权建设，推进城乡和谐社区建设，提高基层民主管理水平和服务水平。扎实做好“双拥”共建工作，巩固和发展军政军民团结。加强民族宗教工作，推进民族团结进步。进一步落实安全生产责任制，狠抓各项措施的落实和安全隐患整治，杜绝重特大事故发生。进一步完善应急工作机制，做好防震减灾工作，加强自然灾害的监测、预警和防控，提高应对和处置突发公共事件的能力；进一步重视和加强信访工作，有效预防和化解各类社会矛盾。推进“五五”法制宣传教育，完善矛盾纠纷调处机制；健全社会治安防控体系，深入开展平安贺州创建活动，依法严厉打击各类违法犯罪活动，维护社会稳定。

保护和改善生态环境。抓好自然保护区建设，进一步加强龟石水库饮用水水源保护和重点旅游景区周边环境、贺江流域、矿山环境、农村环境综合整治。推进珠江流域防护林、生态公益林、退耕还林等生态工程建设。做好地质灾害防治工作。

各位代表！严峻的形势、艰巨的任务对政府工作提出了更高要求。必须加强政府自身改革和建设，增强贯彻落实科学发展观的自觉性和坚定性。坚持依法行政，继续减少和规范行政审批，加强经济调节和市场监管，更加注重履行社会管理和公共服务职能。进一步健全重大工作部署督办、机关绩效考评、依法行政考核、行政效能监察等激励监督机制，推进政务公开，加强电子政务建设。出实招、办实事、求实效，坚决克服形式主义、官僚主义，减少“文山会海”。严格执行党风廉政建设责任制，加强审计督查，切实做到廉政勤政，坚决纠正损害群众利益的不正之风。坚持问计于民、科学决策，进一步健全社情民意的信息反馈机制、重大决策事项社会公示制度和社会听证制度，提高应对困难和风险的能力。

各位代表！今年的经济社会发展任务繁重，使命光荣，责任重大。让我们在市委的领导下，深入贯彻落实科学发展观，坚定信心，齐心协力，迎难而上，奋力拼搏，为实现全市经济社会破位争先作出新的贡献！
